# Supplementary material for: Schizophrenia risk variants modulate transcription factor binding and gene expression in cortical cell types
Source: Cell Mol Life Sci. 2026 Mar 21;83(1):188. doi: 10.1007/s00018-026-06177-2 (PMC13046938; doi:10.1007/s00018-026-06177-2)

Supplemental Information

Supplementary Figures and Legend

Figure S1: Number of peaks and scCREs identified in each cell type. (**a**) Bar plot showing the number of marker peaks identified in each cell type from the single-nucleus ATAC-seq data. (**b**) Bar plot showing the number of scCREs identified in each cell type. Dark blue bars represent the number of peaks called from pseudobulk samples, light blue bars represent the number of peaks accessible in at least 5% of the nuclei, and the total height represents the total number of scCREs, which is the union of both sets.

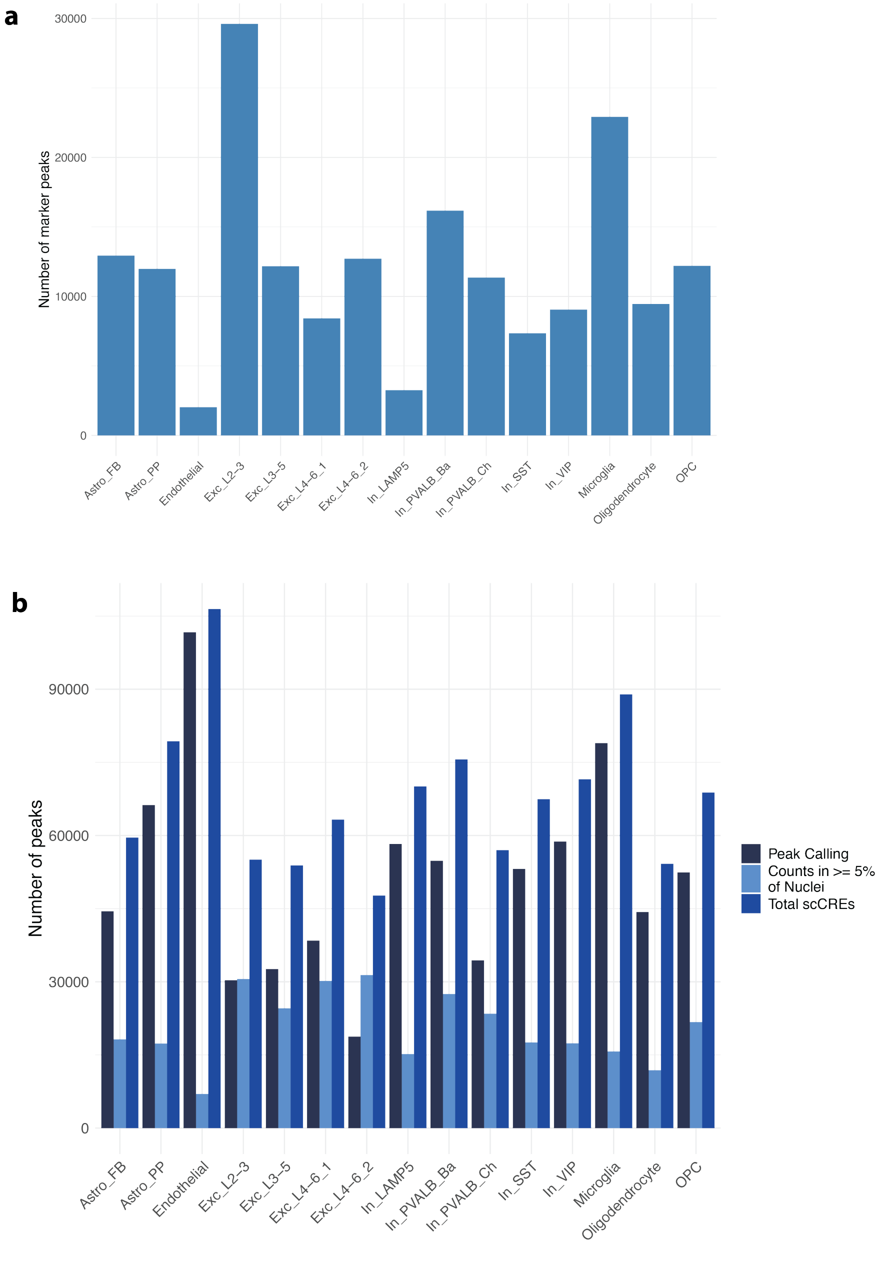

Figure S2: Differential TF binding caused by GWAS SNPs across the genome. Heatmap showing the delta binding scores for individual schizophrenia-associated SNPs across different motifs and cell types. Each row represents a motif, and each column represents a SNP. The color scale indicates the direction and magnitude of the delta binding score (red: gained binding, blue: lost binding). Chromosomes are color-coded.

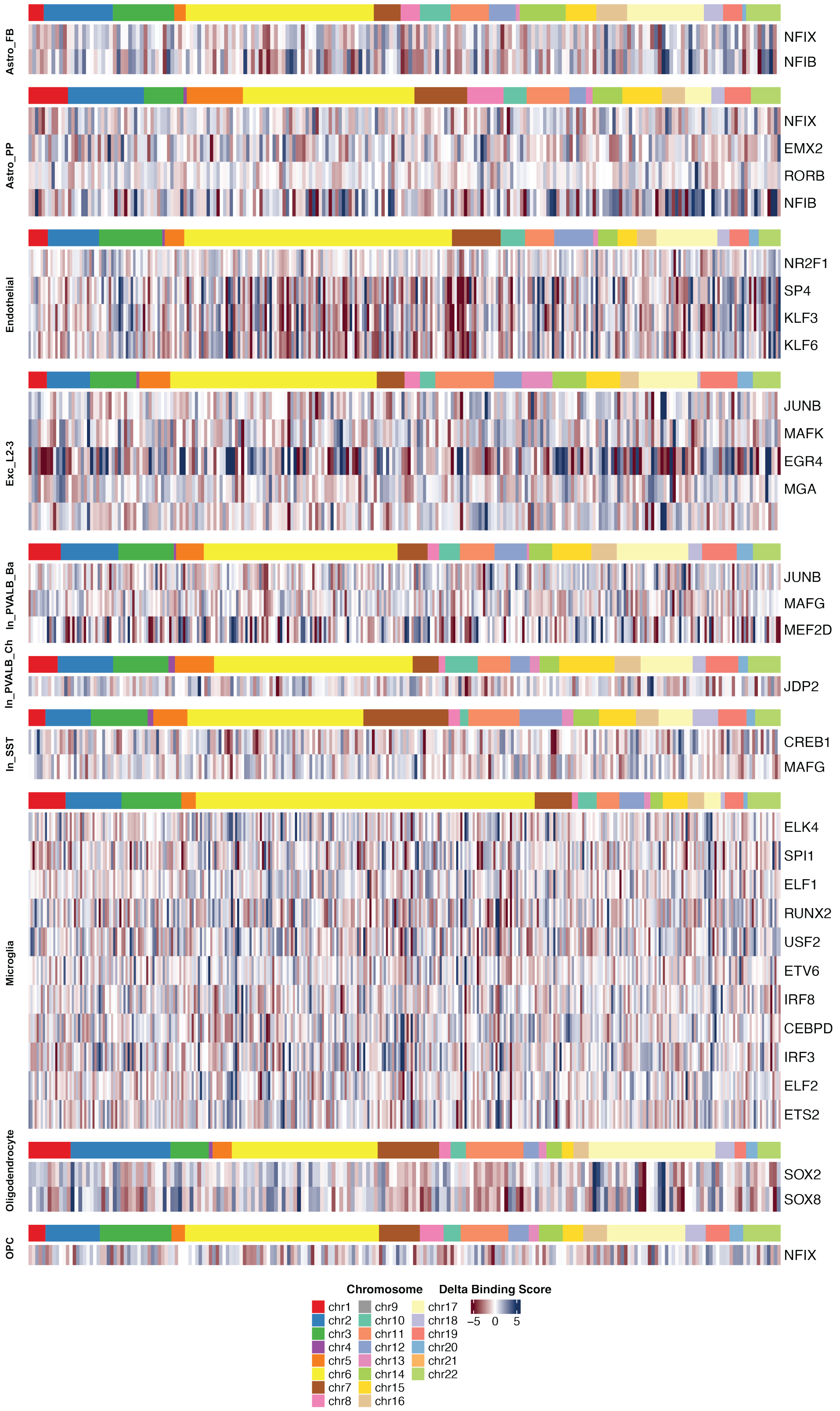

Supplement: Supplementary file 1 — Supplementary file1 (PDF 4630 KB) [file 18_2026_6177_MOESM1_ESM.pdf]
